# Vitamin D Status Predicts 30 Day Mortality in Hospitalised Cats

**DOI:** 10.1371/journal.pone.0125997

**Published:** 2015-05-13

**Authors:** Helen Titmarsh, Scott Kilpatrick, Jennifer Sinclair, Alisdair Boag, Elizabeth F. Bode, Stephanie M. Lalor, Donna Gaylor, Jacqueline Berry, Nicholas X. Bommer, Danielle Gunn-Moore, Nikki Reed, Ian Handel, Richard J. Mellanby

**Affiliations:** 1 Royal (Dick) School of Veterinary Studies and The Roslin Institute, The University of Edinburgh, Roslin, Midlothian, United Kingdom; 2 Specialist Assay Laboratory (Vitamin D), Clinical Biochemistry, Manchester Royal Infirmary, Manchester, United Kingdom; University of Alabama at Birmingham, UNITED STATES

## Abstract

Vitamin D insufficiency, defined as low serum concentrations of the major circulating form of vitamin D, 25 hydroxyvitamin D (25(OH)D), has been associated with the development of numerous infectious, inflammatory, and neoplastic disorders in humans. In addition, vitamin D insufficiency has been found to be predictive of mortality for many disorders. However, interpretation of human studies is difficult since vitamin D status is influenced by many factors, including diet, season, latitude, and exposure to UV radiation. In contrast, domesticated cats do not produce vitamin D cutaneously, and most cats are fed a commercial diet containing a relatively standard amount of vitamin D. Consequently, domesticated cats are an attractive model system in which to examine the relationship between serum 25(OH)D and health outcomes. The hypothesis of this study was that vitamin D status would predict short term, all-cause mortality in domesticated cats. Serum concentrations of 25(OH)D, together with a wide range of other clinical, hematological, and biochemical parameters, were measured in 99 consecutively hospitalised cats. Cats which died within 30 days of initial assessment had significantly lower serum 25(OH)D concentrations than cats which survived. In a linear regression model including 12 clinical variables, serum 25(OH)D concentration in the lower tertile was significantly predictive of mortality. The odds ratio of mortality within 30 days was 8.27 (95% confidence interval 2.54-31.52) for cats with a serum 25(OH)D concentration in the lower tertile. In conclusion, this study demonstrates that low serum 25(OH)D concentration status is an independent predictor of short term mortality in cats.

## Introduction

Vitamin D is traditionally known for its role in calcium homeostasis and bone metabolism. However, it has been demonstrated that numerous types of cells express the vitamin D receptor and it is now clear that the physiological roles of vitamin D extend beyond the maintenance of skeletal health [1, 2]. Vitamin D insufficiency, which is typically assessed by measuring the major circulating form of vitamin D, 25 hydroxyvitamin D (25(OH)D), has been associated with a number of disorders including hypertension [3], diabetes [4], cardiovascular diseases [5], cancer [6], autoimmune conditions [7] and infectious diseases [8–10]. Furthermore, low serum 25(OH)D concentrations have also been linked to all-cause mortality in the general human population [11]. Meta-analyses have demonstrated that serum 25(OH) concentrations are an important predictor of survival in people with a wide variety of illnesses [12–15].

However, interpretation of these studies is challenging. A large number of factors are known to influence serum 25(OH)D concentrations in humans including ethnicity [16–18], diet [18–20], seasonality [21], latitude and exposure to sunlight [22–24], obesity[25, 26], age [27] and gender [28]. Consequently, exploring the relationship between serum 25(OH)D concentrations and all-cause mortality in a model system in which many of these confounding factors are avoided would be of significant interest. Furthermore, a model system which did not require disease to be induced in otherwise healthy animals would allow the number of animals used in scientific research to be reduced. We predict that client owned, domesticated cats which developed spontaneous disease would be a suitable model in which to study the relationship between vitamin D and all-cause mortality. Advantages of investigating the role of 25(OH)D on health outcomes in cats include a more standard dietary intake of vitamin D since almost all cats which attend our referral veterinary hospital eat a commercial diet which is supplemented with a similar amount of vitamin D [29]. In addition, cats do not synthesize vitamin D cutaneously meaning that serum 25(OH)D concentrations are not influenced by exposure to UV radiation [30, 31].

Investigating the role of serum 25(OH)D concentrations and all-cause mortality in cats would be of interest to veterinarians since it is presently difficult to accurately predict mortality in hospitalised, ill cats. The identification of clinical measures which were predictive of mortality would be extremely helpful in providing much needed prognostic information to owners of ill cats. The aim of this study was to investigate whether serum 25(OH)D concentrations was a predictor of short term, all-cause mortality in hospitalised ill cats. We have recently demonstrated that vitamin D metabolism is altered in dogs and cats with a wide range of infectious, inflammatory and neoplastic conditions, highlighting the need to clarify the relationship between serum 25(OH)D concentrations and mortality [32–38]. We hypothesised that cats with low serum 25(OH)D concentrations would have higher mortality at 30 days post admission than cats which were vitamin D replete.

## Material and Methods

### Study Population

Consecutive cats examined at the Royal (Dick) School of Veterinary Studies, Hospital for Small Animals were considered eligible for inclusion in the study. Informed consent for the use of residual clinical blood samples for research purposes was obtained at admission for each cat enrolled. Ethical approval for the study was obtained from the University of Edinburgh’s Veterinary Ethical Review Committee.

Clinical records were reviewed for each cat enrolled. The age, sex and breed were recorded for each cat. Survival data was obtained from clinical records or follow up telephone calls to clients and referral veterinary surgeons for each cat at day 30 post initial presentation. The following clinical information was extracted for each patient: white blood cell count, packed cell volume (PCV), serum albumin, serum creatinine, sodium concentrations, potassium concentrations, total calcium concentration and 25(OH)D concentrations. In addition, the appetite of the cats was graded as normal or reduced. Haematology variables were measured on an ADVIA(r) 2120i System with Autoslide (Siemens Medical Solutions Diagnostics Ltd California, USA). Biochemistry parameters (serum sodium, potassium, creatinine, albumin and total calcium) concentrations were measured on an ILab650 biochemistry analyser, (Diamond Diagnostics, USA).

Following handling of blood samples for routine diagnostic procedures, serum samples were stored initially at -20°C and later moved to -70°C for longer term storage until 25(OH)D concentrations were measured as a batch. Previous studies have indicated that 25(OH)D is stable when stored at -20°C [39]. Serum concentrations of 25(OH)D_2_ and 25(OH)D_3_ were determined by liquid chromatography tandem mass spectrophotometry (LC-MS/MS) using an ABSciex 5500 tandem mass spectrophotometer (Warrington, UK) and the Chromsystems (Munich, Germany) 25OHD kit for LC-MS/MS following the manufacturers’ instructions (intra- and inter-assay CV 3.7% and 4.8% respectively). This Supraregional Assay Service laboratory is accredited by CPA UK (CPA number 0865) and has been certified as proficient by the international Vitamin D Quality Assurance Scheme (DEQAS). Total 25(OH)D is defined as the sum of 25(OH)D_2_ and 25(OH)D_3_. The laboratory measuring the vitamin D metabolites were blinded to clinical data from the enrolled cats. In addition, clinicians were not aware of 25(OH)D results during the clinical management of the cats.

### Statistical Analysis

Initially, we compared the 25(OH)D concentrations between cats which died within 30 days of sampling to cats which survived by a Mann-Whitney U test. In order to investigate for the presence of confounding variables we constructed a standard binary logistic regression model of death by 30 days. We included a range of clinical and biochemical data including sex, age, breed, total white blood cells, packed cell volume and serum concentrations of albumin, total calcium, creatinine, sodium and potassium. We also included an assessment of appetite as a binary variable of normal or reduced. Initially we included 25(OH)D concentrations as a linear predictor within the logistical regression model. We also categorised serum 25(OH)D concentration into 3 categories based on 33% and 66% tertiles treating the variable as a three level factor and also as low versus middle and high categories combined. We used Akaike’s information criteria (AIC—a parameter penalised measure of model fit) to stepwise select variables which were to be retained to identify a final model with minimum AIC (i.e. best parameter penalised fit). P values for individual variables were calculated using Wald’s test. A p-value of < 0.05 was considered to be evidence of statistical significance.

## Results

### Study Population

A total of 99 cats were recruited to the study. The median age of the cats was 96 months. There were 3 entire male, 56 neutered males, 1 entire female and 39 neutered female cats. Breeds included in the study were 62 Domestic Short Hairs, 8 Domestic Long Hairs, 6 Maine Coons, 5 Burmese, 3 Bengals, 2 Tomikense, 2 Siamese, 2 Ragdolls, 2 Burmese crosses, 2 Oriental Short Hair, 1 Manx Cat, 1 British Short Hair, 1 Chinchilla, 1 Burmillia and 1 Abyssinian.

**Fig 1:**
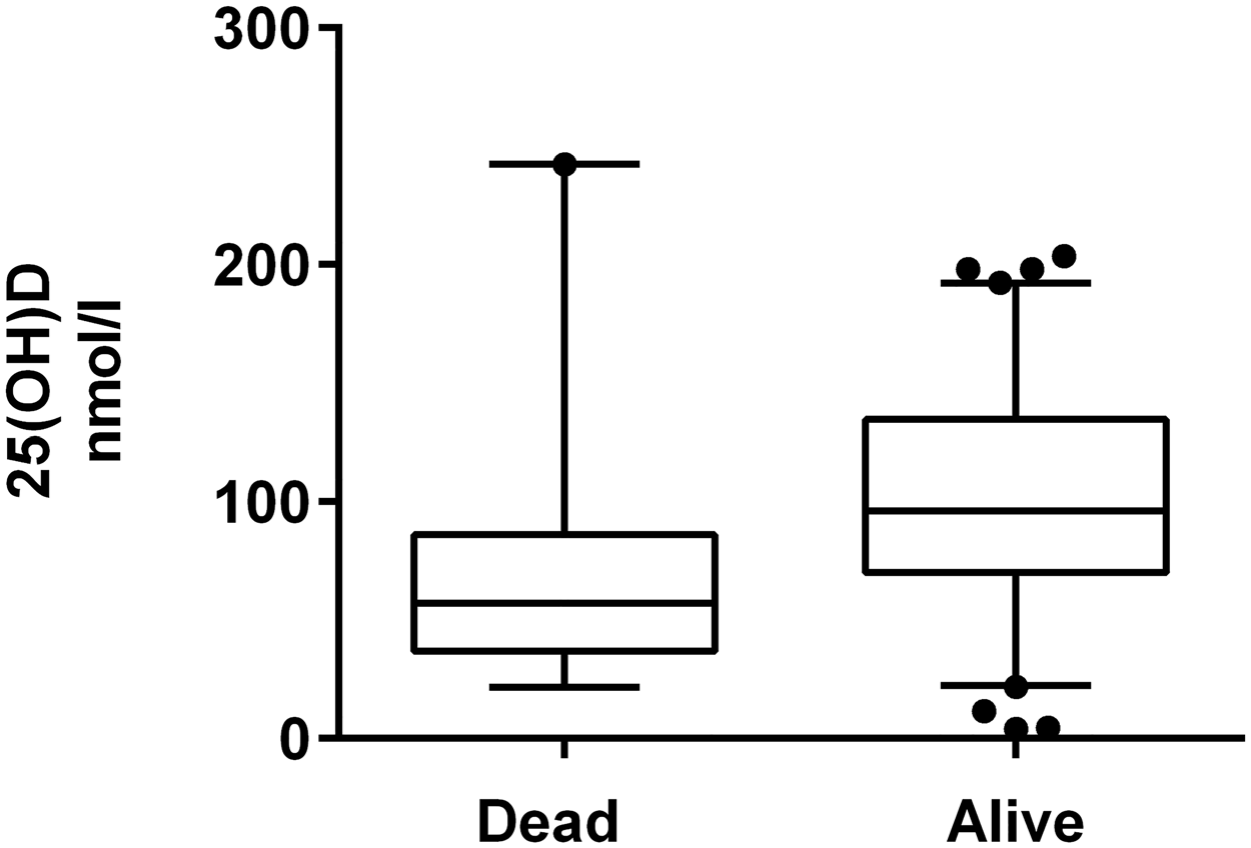
Box and whiskers plot of serum concentrations of 25(OH)D in nmol/l in cats which died or were alive at 30 days post admission. Box extends from 25^th^-75^th^ percentiles with solid line representing the median value. Whiskers extend to 5^th^-95^th^ percentiles.

There was a significant difference between the 25(OH) D concentrations between cats which were alive (n = 80) compared to cats which had died at 30 days (n = 19) (p = 0.0022, Fig 1). Using serum 25(OH) D concentrations as a linear predictor of survival within the logistic regression model, none of the variables, including 25(OH)D concentration, were significant predictors of mortality. Since several studies in humans have also shown a non-linear relationship between vitamin D status and mortality [11, 40, 41], we investigated whether serum 25(OH)D concentrations were a significant predictor of mortality when represented as a categorical variable. We found that cats with a 25(OH)D concentration in the lower tertile had an increased risk of mortality compared to cats in the middle tertile reference category (Table 1). There was no significant difference in survival between cats in the upper and middle tertile (Table 1). The only other parameters which were associated with an increased risk of mortality by 30 days were potassium concentration and a reduced appetite (Table 1).

**Table 1:** Results of logistic regression model including serum 25(OH)D concentration as three tertile categorical variable.

Variable	Odds ratio (95% CI)	P value
**Potassium**	4.23 (1.36–14.59)	0.0153
**Reduced appetite**	4.05 (1.17–17.04)	0.0370
**25(OH)D category low (<73.6nmol/l)**	9.51 (2.25–57.07)	0.0051
**25(OH)D category middle (73.6–110.05nmol/l)**	Reference category	Reference category
**25(OH)D category high (>110.05nmol/l)**	1.31 (0.21–8.66)	0.7681

Table only shows significant variables. (AIC = 85.50)

Based on the results of the three tertile model and the epidemiological data which links low vitamin D status to poor health outcomes [12], we combined the middle and upper tertile into a single binary predictor in a third model. A serum 25(OH)D concentration in the lower tertile remained predictive of 30 day mortality (Table 2). Again, we found that potassium concentration and a reduced appetite were the only other parameters included in the third model which were predictive of survival (Table 2).

**Table 2:** Results of logistic regression model combining vitamin D as a categorical variable using 25(OH)D as a binary predictor of lower tertile versus middle and upper tertiles combined.

Variable	Odds ratio (95% CI)	P value
**Potassium**	4.14 (1.34–14.21)	0.0161
**Reduced appetite**	4.02 (1.16–16.83)	0.0379
**25(OH)D category low (<73.6nmol/l)**	8.27 (2.54–31.52)	0.0008

Table only shows significant predictors. (AIC = 83.58)

## Discussion

The central finding of this study demonstrates that hospitalised ill cats with low serum 25(OH)D concentrations were less likely to survive 30 days. Using a regression model which included serum 25(OH)D concentrations as a linear variable, none of the 12 clinical, biochemical and hematological parameters, including 25(OH)D concentrations, were predictive of mortality. However, when we performed a second analysis in which we included serum 25(OH)D concentrations as a categorical variable, we found that low vitamin D status was an independent predictor of short term mortality.

The finding that there was a relationship between low serum 25(OH)D concentrations and mortality is consistent with numerous human studies [13, 42, 43]. In addition, the observation that there was not a linear relationship between vitamin D status and survival is also consistent with studies in human patients [11, 40]. Several human studies have reported that there is minimal benefit of having high serum 25(OH)D concentrations and a number have linked high vitamin D status to negative health outcomes [40, 44].

There are a wide range of potential mechanisms by which low vitamin D status may influence health outcomes. The vitamin D receptor is expressed on many immune cell types and it is clear that vitamin D can modulate both the innate and acquired immune responses via effects on monocytes, macrophages, dendritic cells and lymphocytes [45, 46]. Vitamin D has also been shown to profoundly modulate pro-inflammatory responses [47, 48]. The pleiotropic extra-skeletal effects of vitamin D also extends to vascular function [49] and cellular proliferation and differentiation [50]. Supplementation with vitamin D has also been shown to reduce pro-inflammatory cytokines in patients with cardiovascular disease [51]. The renin-angiotensin system is also negatively regulated by vitamin D [52, 53]. Up-regulated renin-angiotensin activity is associated with systemic hypertension, renal dysfunction, vascular damage [54] and cardiac hypertrophy [55]. Vitamin D is also inversely associated with parathyroid hormone, although this change is not seen in all patients with hypovitaminosis D [56], and excess parathyroid hormone has been related to increased risk of heart failure [57]. All of these diverse effects of low vitamin D concentrations may impact on survival in domesticated cats.

Our study also demonstrated that reduced appetite was an independent predictor of short term mortality in cats. This finding is similar to human studies where reduced appetite has been linked to poor health outcomes in elderly patients [58]. However, serum 25(OH)D concentrations remained a significant predictor of mortality when the results were corrected for reduced appetite. This suggests that the association between low serum vitamin D concentrations and mortality is not simply due to reduced dietary intake of vitamin D in hospitalised cats.

The study also demonstrated that potassium concentrations were linked to mortality with increasing potassium concentrations associated with poor survival outcomes. High serum potassium concentrations have been associated with mortality in critical care patients, even when increases in potassium are modest [59], and in patients with cardiac and renal disease [60]. The mechanism(s) by which hyperkalemia influences mortality are unclear. However raised potassium concentrations can result in altered neurological, cardiac and muscular function [59]. Furthermore, declining renal function is also associated with hyperkalemia [61] and hyperkalemia has been shown to be associated with serious infections and haemorrhage [59] which may in part explain its association with mortality.

In contrast to human medicine, little is known about the factors which are involved in all-cause mortality in cats. Previous studies have focused particularly on cats admitted to an intensive care unit (ICU), rather than across a wider hospital population [62, 63]. Therefore, the use of vitamin D to predict survival in a general hospital population is an important feature of this study. A previous reported predictor of feline survival is the Feline Acute Patient Physiologic and Laboratory Evaluation (Feline APPLE) Score [63]. This scoring system has been validated only for feline ICU patients and requires several clinical and diagnostic parameters to be assessed. A univariable measure such as serum 25(OH)D concentration may provide a simpler and more readily usable predictor of mortality.

It cannot be concluded that serum 25(OH)D is causally linked to mortality from our finding that low vitamin D status is an independent risk factor of 30 day mortality in hospitalized, ill cats. This would require further prospective studies, including randomized, placebo controlled supplementation studies of cats with low vitamin D status. In light of our finding that cats with 25(OH)D concentrations in the upper tertile had a similar incidence of mortality as cats in the middle tertile, future studies should focus on assessing whether correction of hypovitaminosis D improves health outcomes. This approach is supported by observations from human trials in critically ill patients [64]. Similarly, a study investigating the effects of vitamin D on cardiovascular morbidity and mortality, revealed that although supplementation improved overall survival, the effects were only significant in vitamin D deficient patients [65].

In conclusion, this study supports the hypothesis that low serum vitamin D status is predictive of 30 day mortality in hospitalised cats. The finding that low serum 25(OH)D concentrations are negatively correlated with survival supports the initiation of follow up clinical trials to examine the influence of vitamin D supplementation on disease outcome. Our study also indicates that domesticated cats with spontaneous illnesses may provide a valuable alternative to rodent models in which the effects of vitamin D on health outcomes can be probed without the need to induce disease in otherwise healthy animals.
